# Discontinuously supervised aerobic training vs. physical activity promotion in the self-management of type 2 diabetes in older Italian patients: design and methods of the ‘TRIPL-A’ randomized controlled trial

**DOI:** 10.1186/s12877-018-1022-x

**Published:** 2019-01-11

**Authors:** Francesco Lucertini, Carlo Ferri Marini, Davide Sisti, Vilberto Stocchi, Ario Federici, Franco Gregorio, Donata Piangerelli, Carlos Chiatti, Antonio Cherubini, Massimo Boemi, Fabio Romagnoli, Michela Cucchi, Federica D’Angelo, Maria Paola Luconi, Anna Rita Bonfigli

**Affiliations:** 1 Department of Biomolecular Sciences – Division of Exercise and Health Sciences, University of Urbino Carlo Bo, Via I Maggetti, 26/2 –61029 Urbino, PU Italy; 2 Department of Biomolecular Sciences – Unit of Medical Statistic and Biometry, University of Urbino Carlo Bo, Piazza Rinascimento, 7–61029 Urbino, PU Italy; 3 Department of Biomolecular Sciences, University of Urbino Carlo Bo, Via I Maggetti, 26/2–61029 Urbino, PU Italy; 4 ASUR Marche – Diabetology Unit, Via Montello, 4–60035 Jesi, Italy; 5 IRCCS INRCA, Scientific Direction, Via della Montagnola, 81–60127 Ancona, Italy; 6 IRCCS INRCA, Geriatria, Accettazione Geriatrica, Centro di Ricerca per l’Invecchiamento, Via della Montagnola, 81–60127 Ancona, Italy; 7 IRCCS INRCA, Diabetology Unit, Via della Montagnola, 81–60127 Ancona, Italy

**Keywords:** Diabetes mellitus type 2, Exercise training, Sedentary lifestyle, Habits, Older, Quality of life, Randomized controlled trial, Patient compliance, Exercise referral scheme, Computer-based intervention

## Abstract

**Background:**

Physical activity (PA) has health benefits for people with type 2 diabetes (T2D). Indeed, regular PA is considered an important part of any T2D management plan, yet most patients adopt a sedentary lifestyle.

Exercise referral schemes (ERS) have the potential to effectively promote physical activity among T2D patients, and their effectiveness may be enhanced when they are supported by computer-based technologies.

The ‘TRIPL-A’ study (i.e., a TRIal to promote PhysicaL Activity among patients in the young-old age affected by T2D) aims to assess if realizing an innovative ERS, based on a strong partnership among general practitioners, specialist physicians, exercise specialists, and patients, and supported by a web-based application (WBA), can effectively lead sedentary older T2D patients to adopt an active lifestyle.

**Methods:**

A randomized controlled design will be used, and an ERS, supported by a WBA, will be implemented. 300 physically inactive T2D patients (aged 65–74 years) will be assigned to either an intervention or control arm. Control arm patients will only receive behavioral counseling on physical activity and diet, while intervention arm patients will also undergo an 18-month (3 day/week), discontinuously supervised aerobic exercise training program. The trial will be divided into six three-month periods: during first, third and fifth period, an exercise specialist will supervise the training sessions and, using the WBA, prescribe exercise progression and monitor exercise adherence. Patients will exercise on their own in the other periods.

Patients’ sedentary behaviors (primary outcome), PA level, fitness status, metabolic profile, psychological well-being, quality of life, and use of health care services (secondary outcomes) will be assessed at baseline and at 6, 12, and 18 months from baseline.

Repeated measure ANCOVAs will be used to compare the intervention and control arm with respect to each study outcome measure.

**Discussion:**

Primary and secondary outcome results will allow us to evaluate the effectiveness of an ERS, specifically designed for the management of T2D clinical conditions and supported by a WBA, in promoting PA within Italian primary care settings.

**Trial registration:**

This trial is retrospectively registered under the Australian New Zealand Clinical Trials Registry (reference number: ACTRN12618001164280; registered 13 July 2018).

## Background

The baby boomer generation, one among the largest cohorts of history, is approaching retirement age and the maintenance of the health status and independence of this aging segment of the population (i.e., ≥ 65 years) is currently a major public health concern [1].

Regular physical activity (PA) is one of the main predictors of health and well-being in older people, whereas sedentary behaviors and physical inactivity are associated with an increased risk of over 20 health conditions, including heart diseases, cancer, stroke, and diabetes [2]. Indeed, PA has proven positive effects on people who have stable chronic diseases, such as diabetes, helping to reduce the risk of disability and the use of health care services. Structured PA is now considered an important part of type 2 diabetes (T2D) management plan [3] since it improves blood glucose control and well-being, while reducing diabetes-related complications and cardiovascular risk factors. In addition, reducing sedentary behavior and interrupting prolonged sitting are also recommended to improve blood glucose levels in adults with T2D [4] and such measure have come to be viewed as critical to effective T2D management [5]. In fact, reducing daily sitting time has been shown to yield marked improvements of diabetes clinical goals [6], even when PA engagement is accounted for.

However, despite the well-known benefits of PA and reduced sitting time, most older people adopt a sedentary lifestyle and evidence-based interventions promoting PA largely fail to be translated into practice [7, 8]. This is particularly true for T2D patients, who show low levels of physical activity, high levels of sedentary behaviors [9], and a disappointing low participation rate in diabetes education classes [10]. Indeed, long-term diabetic education interventions do not often yield significant improvements in blood glucose control, regardless of whether they are peer-led in community-based settings [11] or administered by previously trained general practitioners (GP) in clinical settings [12, 13]. Furthermore, existing studies on this topic mostly focus on middle-aged patients, while the literature on older T2D patients is scant (see [14] for a review).

Poor integration among the stakeholders in disease prevention is one of the major causes of the low effectiveness of PA promotion [15]. The literature has shown that exercise referral scheme (ERS) interventions – in which health care professionals refer patients who will benefit from PA to third party services that prescribe and monitor an exercise program tailored to their individual needs – may represent an effective model to promote PA in primary care settings [16, 17]. Specifically, a report from the UK National Institute for Health Research [18] clearly shows that primary care-based ERSs designed for T2D patients lead to a significant improvement in glucose control and a reduction in diabetes complications compared to a non-active intervention comparator. Promoting regular PA in older T2D patients could therefore represent a cost-effective strategy to improve the management of their health condition and their quality of life, while reducing the burden of health care resources. Indeed, findings from other studies (e.g., see [19, 20]) suggest that patients that are referred to PA by GPs are more likely to be active than those who are referred by a visiting specialist. Interestingly, studies have also suggested that the effectiveness of interventions aimed at promoting PA, in both healthy adults [21, 22] and T2D patients [23, 24], may be enhanced when traditional methods are supported by the use of web-based applications (WBA).

To our knowledge, long-term studies implementing an ERS supported by web-based technologies aiming to improve the management of the clinical conditions of older T2D patients have never been conducted in Italy.

## Methods

### Objectives

The ‘TRIPL-A’ study (i.e., a TRIal to promote PhysicaL Activity among patients in the young-old age affected by type 2 diabetes) aims to assess if realizing an innovative ERS based on a strong partnership among GPs, specialist physicians, exercise specialists, and patients, and supported and facilitated by a multi-platform WBA, can effectively lead older sedentary T2D patients to adopt an active lifestyle.

We hypothesize that this ERS will be able to reduce inactivity time and the use of healthcare resources.

Accordingly, the primary objective of the trial is to determine if the ERS reduces sedentary behaviors, while the secondary objectives are to determine if it increases the patient’s physical activity level, fitness status, metabolic profile, psychological well-being, and quality of life, while reducing the use of health care services.

### Design

**Fig. 1:**
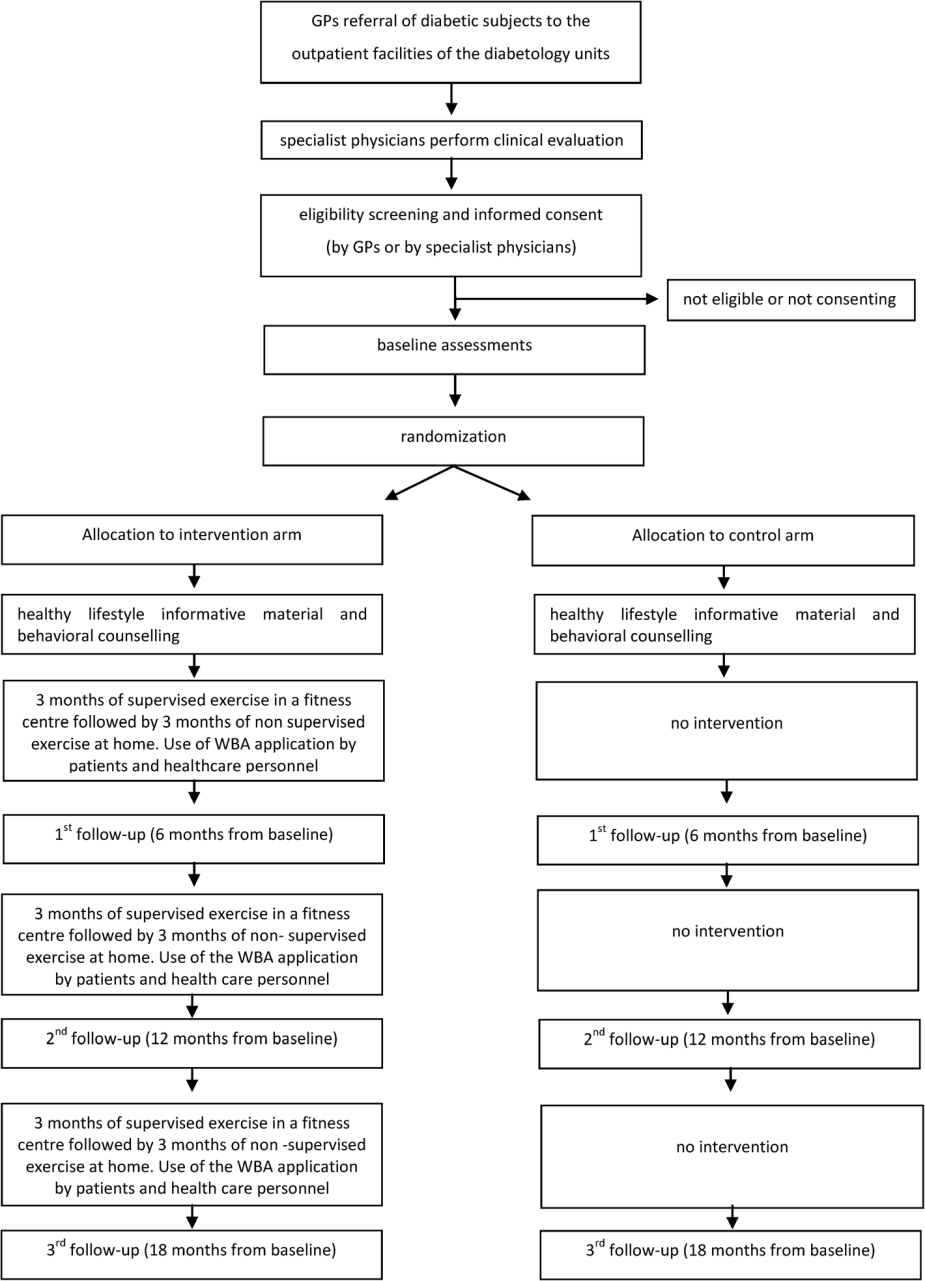
Study design

The ‘TRIPL-A’ is a randomized, controlled, open, bicenter trial, with two parallel groups. The study will be carried out in two urban areas (independent in terms of staff, facilities and participants) in the Marche region (Central Italy): the Diabetology Unit of the IRCCS-INRCA Hospital in Ancona and the Diabetology Unit of the ASUR Hospital of Fabriano (AN). Enrolled patients will be assigned to either the intervention or control arm. The assessments will be performed within the medical facilities, while the exercise training program will be carried out within third party fitness facilities. An overview of the study is shown in Fig. 1.

### Participants’ eligibility

Older T2D patients who meet the inclusion criteria and wish to participate voluntarily will have to sign a written informed consent form before being recruited and any trial procedure occurs. No procedures will be performed until the patient has signed the consent form and been accepted into the trial. Only recruited patients who do not meet any of the exclusion criteria will be enrolled and will participate in the trial.

Trial staff must withdraw enrolled patients at any time if they no longer meet the inclusion and exclusion criteria.

#### Inclusion criteria

Patients who meet all of the following criteria will be recruited:

- age range (years): 65 to 74;

- suffering from T2D according to ADA criteria [25];

- physically inactive or insufficiently active (i.e., level 1 or level 2 exposure to physical inactivity) according to the WHO framework of health risks quantification [26], as assessed using the results of the *International Physical Activity Questionnaire* (IPAQ) short form.

#### Exclusion criteria

Patients who will meet one or more of the following criteria will not be enrolled:

- receiving *β*-blockers therapy or any other drug that could affect heart rate, either at rest or in response to aerobic exercise;

- suffering from chronic obstructive pulmonary disease;

- terminally ill (life expectancy < 6 months);

- suffering from severe cardiovascular disease (including New York Heart Association class III or IV congestive heart failure), clinically significant valvular disease, history of cardiac arrest, presence of an implantable defibrillator, or uncontrolled angina that would interfere with the ability to participate fully in either study arm;

- history of myocardial infarction, transient ischemic attack or stroke in the six months before the recruitment date;

- suffering from any condition that, in the clinical judgement of the principal investigator, might harm the patient if he or she were to participate in the trial.

### ERS and WBA implementation

The ERS to be used in the present study is a physical activity promotion and adoption framework delivered through primary care institutions and implemented within third party facilities. The ERS starts with a GP referral of the eligible T2D patient to the specialist physician of the Diabetology Unit, who will assess her/his clinical status and, in turn, refers the patient to the exercise specialist of the third party facility. The exercise specialist will design a personalized exercise program based on the patient’s medical needs and her/his clinical status, both of which will be provided by the specialist physician. The ERS will also provide feedback through a WBA specifically designed for the study, which will allows the specialist physician and the GP to monitor the adherence of each patient to the exercise prescription made by the exercise specialist.

The WBA will be developed and made available on the Web to patients and trial staff. The WBA will be designed to serve as both the framework on which the ERS is based, and the platform where data are recorded, stored, processed and used by the investigators for the purposes of the trial. Above all, the WBA is designed to enhance and foster the partnership among the study stakeholders, who will have different roles and be based in different locations. In fact, its web-based nature will allow the different stakeholders to access, via a web-browser (therefore regardless the the device used and/or its form factor), to the contents made available according to the privileges granted to each user. All the enrolled patients will have access to generic pages promoting an active lifestyle, providing physical activity and healthy diet guidelines and other useful information on how to self-manage T2D in older adults. Patients enrolled in the intervention arm will also be able to access data generated from their own training (such as adherence to the protocol, average aerobic exercise intensity and duration, motivational feedback based on their training results, etc.). Those data will be made available by the WBA after the automatic processing of the exercise training data entered by the exercise specialists, who will have read and write privileges for all the non-clinically relevant information and all the exercise session report forms of the enrolled patients. GPs and specialist physicians will have access to both the clinical, non-clinical and exercise session data of all the patients they refer to the trial. They will therefore be able to monitor the patient’s adherence to the trial (according to the specific requirements of the study arm to which the patient has been assigned) and readily obtain the information needed to give the patient motivational feedback to improve compliance. The WBA will also allow investigators to store and access patients’ clinically and non-clinically relevant data (copies of patients’ case report forms, test results, and compliance reports; timeline of the trial; randomization; etc.) and to set the range of values of the exercise prescription parameters of the whole training intervention. The WBA will use both the parameters of the exercise prescription and those resulting from each training session in order to constantly provide an updated profile of each patient in the intervention arm. The profile can then be used by the exercise specialists to adjust the exercise type, duration and intensity for the subsequent training session. Finally, the principal investigator will have full privileges, including the ability to export all the data stored in the WBA online database in files to be imported into statistical software.

#### Exercise referral and recruitment strategies

When making referrals, participating GPs and specialist physicians will first screen their patients using the inclusion criteria and then send their contact details to the trial staff for enrollment.

The following promotional initiatives will be implemented:

- providing GPs working near the trial areas of the trial with informative material on healthy lifestyle (see below) and asking them to participate in the trial by adopting the ERS; participating GPs will then provide their T2D patients with information on the trial and refer them to the TRIPL-A;

- distributing fliers in the clinical centers and facilities involved in the trial;

- displaying posters in the clinical centers and facilities involved in the trial.

#### Healthy lifestyle informative material

TRILP-A staff will create and make available an “active lifestyle leaflet”, whose purpose is to promote PA in older T2D patients, and a “healthy eating brochure”, whose purpose is to help T2D patients to improve their dietary habits. For this purpose infographic materials from the “Handbook for Canada’s physical activity guide to healthy active living” [27] will be translated into Italian and modified according to the needs of this trial.

The healthy eating brochure will help diabetic patients to improve their dietary habits by balancing meal nutrients composition and making healthy food choices.

### Enrollment and arm assignment

A total of 300 older T2D patients will be enrolled and randomly assigned to either the control or the intervention arm with a 1:1 ratio, using a computerized permuted blocks randomization. The randomizations will be performed using a code created by the statisticians of the INRCA Biostatistical Center, who will not be involved in any other part of the trial. Allocation concealment will be assured because the trial staff will not know the randomization code and procedures (e.g., block sizes), and the allocation will be made only after the termination of all baseline tests (see Fig. 1).

Due to the obvious differences between the interventions (see below), neither staff nor participants will be blinded to group allocation. However, before data analysis, patient allocations and identification numbers will be replaced by categorical variables whose meaning will be unknown to data analysts.

### Intervention

Trial intervention will last 18 months during which time concomitant care will be permitted. Patients of both trial arms will be retained as long as they meet the inclusion and exclusion criteria.

#### Intervention arm

Patients assigned to the intervention arm will receive the healthy lifestyle informative material and a behavioral counselling on physical activity and diet by a trained physician. The healthy lifestyle informative material will be given to the patients during the behavioral counseling and discussed.

A structured exercise program of 18 months, with alternating three-month periods of supervised and non-supervised training (i.e., discontinued supervision), will be implemented. Participants will train three times per week, with at least 48 h between each exercise session.

During the 3 three-month periods of supervised training (period 1: 1st to 3rd month; period 3: 7th to 9th month; period 5: 13th to 15th month), patients will exercise at a fitness center under the supervision of an exercise specialist, who will use the WBA to constantly monitor and update the exercise prescription. In the non-supervised training periods (period 2: 4th to 6th month; period 4: 10th to 12th month; period 6: 16th to 18th month) participants will train on their own and use the WBA to access their aerobic training schedule and parameters (duration and intensity). They will also be asked to enter the actual workout performed into the WBA at the end of each self-monitored exercise session.

The aerobic exercise training protocol will be designed according to the internationally accepted recommendations and guidelines on health-enhancing PA for older adults [28–30] and T2D patients [31–34].

Aerobic exercise intensity will be prescribed and monitored according to heart rate (HR), which is a reliable parameter in the present trial since patients will not be taking any medication that could affect it. For each patient, the percentage of target HR (%HR_target_) prescribed for a given exercise period will be applied to the HR reserve (HRR), i.e. the difference between resting HR (HR_rest_) and maximum HR (HR_max_). Patients’ HR_rest_ will be directly measured, whereas the HR_max_ will be estimated according to the equation proposed by Gellish et al. [35]. For each patient, the exercise session target HR (HR_target_) will then be calculated (in beats per minute) as follows: HR_target_ = [(HR_max_ – HR_rest_) x %HR_target_] + HR_rest_. This approach, namely the “HRR method” [36], is widely accepted as the most accurate in establishing a HR_target_ because, i) it accurately reflects the percentages of the reserve values of oxygen uptake (*V̇*O_2_R), i.e. the gold standard for assessing the relative metabolic intensity, and ii) it allows the correction of the exercise intensity for the HR_rest_, which can be highly variable among subjects [37].

#### Supervised exercise

Aerobic exercise intensity and duration will gradually increase throughout each three-month period (see Table 1).

**Table 1:** Supervised aerobic training rate of progression for exercise session intensity and duration parameters

	Period 1	Period 3	Period 5
Intensity (%HRR range)	40–50	45–55	50–60
Duration (minute range)	20–30	30–40	40–50

Notes: Values are indicative and will be modified according to patient’s response to exercise; %HRR, heart rate reserve percentage

At the end of each session, the exercise specialist will prescribe the exercise duration and intensity of the next session, aiming for a progression rate that will allow patients to reach the upper limit range of each three-month period at least one week before its end. The WBA will help the exercise specialist to implement the progression rate by highlighting: 1) when the patient has completed two consecutive training sessions; 2) the duration and intensity of the previous sessions; 3) the pattern of the previous increases in duration and intensity (in order to alternate the type of increment); 4) the type of exercise (e.g. treadmill, bike, etc.) performed in the previous sessions (which will also be alternated).

The *10-point category ratio scale* (CR-10) of perceived exertion [38] will also be also used to monitor exercise intensity. In order to familiarize participants with the scale, the exercise specialist will 1) provide them with standardized instructions on how to use it, and 2) anchor the upper limit of the scale to a specific feeling experienced in the past during intense PA sessions (i.e., anchoring memory procedure).

### Non-supervised exercise

Aerobic exercise intensity and duration achieved at the end of each supervised three-month period will be used as a reference for the subsequent non-supervised period (see Table 2). Patients will choose the type of aerobic exercise independently but will be required to adhere to the exercise intensity and duration prescribed for that particular three-month period. When patients are not be able to monitor their own exercise HR while exercising, they will be required to control for exercise intensity by trying to achieve the same rate of perceived exertion of the last session of the previous supervised training period. They will also be asked to enter exercise duration, average HR and perceived exertion into the WBA after each training session.

**Table 2:** Non-supervised aerobic training rate of progression for exercise session intensity and duration parameters

	Period 2	Period 4	Period 6
Intensity (%HRR target)	50	55	60
Duration (minutes)	30	40	50

Notes: %HRR, heart rate reserve percentage

#### Control arm

Patients assigned to the control arm will only receive the healthy lifestyle informative material and a behavioral counselling session on physical activity and diet by a trained physician. The healthy lifestyle informative material will be discussed with the patients and handed out to them during the behavioral counselling session.

### Intervention adherence

Three main strategies will be used to improve intervention adherence throughout the trial. Firstly, the healthy lifestyle informative material will be handed out and discussed with all participants at every follow-up. Secondly, periodic feedback (about twice during each three-month period) will be given to the intervention arm participants (by e-mail and/or by telephone). The feedback will be based on training adherence data retrieved from the WBA: acceptable adherence will be reinforced, whereas participants whose adherence has decreased will be provided with motivational inputs. Lastly, intervention arm patients will be contacted by phone each time they attend one fewer session per week than expected for two consecutive weeks.

### Assessments/outcomes

Patients’ anthropometrics, sedentary habits, PA level, fitness status, metabolic profile, psychological well-being, quality of life, and use of health-care resources will be assessed, along with other routine assessments, on four separate occasions: at baseline and at 6, 12, and 18 months from baseline (see Table 3). The difference between the intervention and control arms at the 12-month time-point will be considered the main outcome for each measure.

**Table 3:** Timing of study assessments

	Screening	Visit 1 baseline	Visit 2 follow-up 1	Visit 3 follow-up 2	Visit 4 follow-up 3
Weeks from randomization	-2	0	24	48	76
Visit window in weeks	–	+ 2	+/− 2	+/− 2	+/− 2
Informed consent	X				
Demographics	X				
Inclusion/exclusion criteria	X	X			
Anthropometric measures		X	X	X	X
Clinical variables		X	X	X	X
Randomization		X			
Lifestyle counseling		X			
Lifestyle informative material		X			
WBA training (only for intervention group)		X			
Blood pressure		X	X	X	X
Registration of laboratory assays		X	X	X	X
LDCW		X	X	X	X
IPAQ (short form)		X	X	X	X
EQ-5D-5L		X			X
PSQI		X			X
Healthcare resource consumption		X	X	X	X
Events					
Adverse events including hypoglycemia		X	X	X	X
New diagnoses		X	X	X	X
Intervention compliance check			X	X	X
Trial completion					X

Notes: *LDCW* long distance corridor walk, *IPAQ* International Physical Activity Questionnaire, *EQ-5D-5L* Euro Quality of Life 5 Dimensional questionnaire, *PSQI* Pittsburgh Sleep Quality Index

#### Primary outcome measure

Sitting time will be retrieved from the results of the IPAQ short form and computed as average daily minutes of sitting calculated over a one-week period. The IPAQ is a well validated tool [39–43] for assessing both habitual sitting time and physical activity engagement. The IPAQ short form will be interview-administered by trained personnel.

#### Secondary outcome measures

##### Physical activity level and fitness status

Metabolic Equivalents (MET) per week (an index of physical activity level) will be calculated using the IPAQ short form. Maximal oxygen uptake (*V̇*O_2max_; an index of fitness status) will be calculated using the results of the Long Distance Corridor Walk (LDCW) test and expressed as the sex- and age-specific reference percentile for *V̇*O_2_max [34]. The LDCW has been validated as a reliable shuttle test to estimate *V̇*O_2max_ in subjects between 60 to 91 years of age [44, 45]: after a warm-up of two minutes, the participant is asked to walk 10 laps “as quickly as possible”, over a 20-m course marked with cones (i.e., 400 m).

#### Quality of life

Quality of life will be evaluated with the The *EuroQol 5-Dimensional questionnaire* (EQ-5D-5L), which consists of questions related to five health domains (pain, mood, mobility, self-care and daily activities), yielding an overall quality of life score. Participants’ opinion on their own current health status will also be evaluated using a visual analogue scale ranging from 0 (worst imaginable health status) to 100 (best imaginable health status). The EQ-5D-5L is a widely accepted and standardized tool to measure health-related quality of life in diabetic subjects [46].

#### Healthcare resources

Healthcare resources consumption will be evaluated using a tailored questionnaire, specifically designed for the study to assess direct medical costs of: outpatients visits to specialist physicians, hospital admissions, emergency room visits, use of homecare services, visits to GPs, and use of medications and laboratory examinations.

#### Metabolic profile

Blood fasting glucose and glycated hemoglobin (HbA1c) analyses will be used as indices of glycemic control. Since these tests are routinely performed on diabetic patients, they will be retrieved from the patients’ medical records, selecting the test that was performed closest to each assessment time-point.

#### Sleep disorders

Sleep disorders will be evaluated using the Pittsburgh Sleep Quality Index (PSQI) global score [47] (in which a score > 5 indicates poor sleep). The PSQI is a widely accepted tool to measure sleep-wake disturbances in diabetic patients. It assesses sleep quality, sleep latency, sleep duration, sleep efficiency, sleep disturbances, use of sleep medications, and daytime dysfunction [48].

#### Anthropometry

Height (head in the Frankfurt plane) and weight (light clothes, without shoes) will be measured with the patient standing and body mass index (BMI) will be calculated (kg/m^2^). Waist and hip circumference will be assessed using standard methods (as the average of triplicate measures) and waist-to-hip ratio (WHR) will be calculated.

#### Routine assessments

Systolic (SBP) and diastolic (DBP) blood pressure (mmHg) will be measured with the patient sitting on a chair with back support, while HR_rest_ will be recorded as the average of the last minute out of 10 min of supine rest [49].

### Research staff training

The medical personnel will receive specific training on how to approach to and communicate with the patients to maximize the enrollment rate throughout the recruitment process of the trial. The training will provide the healthcare professionals with standardized information that they can share with the patients, fostering effective communication, particularly as regards the trial aims, allocation procedure and intervention protocol.

All the investigators will undergo several training sessions specifically designed to familiarize them with the timing and protocols of all the trial assessments, and the correct procedures to enter and store patients’ data in the WBA.

Exercise specialists will receive training on how to implement and monitor the exercise prescription in the patients of the intervention arm and on how to input data and query the exercise session database within the WBA. They will be also informed about the risks associated with exercise in older T2D patients and trained on how to recognize the signs and symptoms that call for the immediate termination of the exercise session [34].

### Patients training

All patients will receive training on how access the WBA, retrieve their own data, and enter lifestyle information. Patients of the intervention arm will also be instructed how to enter data of exercise sessions not performed under the supervision of the exercise specialists into the WBA.

### Additional information on data collection, management, and retention

The information collected during the trial will be depersonalized and password-protected, and access to data will be restricted to the TRIPL-A staff in accordance with current Italian law (art 13 D.lgs.196/2003). The complete final dataset will contain no identifying participant information and its access will only be granted to TRIPL-A principal investigators.

Paper-based CRFs will be used to record the results of patient assessments at each time-point (see Fig. 1). CRFs data will also be entered into the WBA to provide the information required to design an exercise prescription tailored to the specific needs of each patient throughout the trial. This will also provide the trial with both a data accuracy verification procedure (the WBA will warn the user when data entry is incorrect or not physiologically plausible and when missing data are missing) and an electronic back-up.

### Statistical methods

#### Sample size calculations

The sample size has been calculated to test the hypothesis of no difference in sitting time per day between intervention and control arms at the 12-month time-point (2-sided independent sample t-test; statistical power 0.8; α level of significance 0.05; 1:1 ratio between intervention and control arm). Given an expected mean value of sitting time of 500 ± 200 min per day [39, 41–43], a sample size of 112 participants in each arm will be necessary to detect a difference of 75 min per day of mean sitting times between intervention and control arm. Therefore, it was assumed that a sample size of 300 participants, i.e. 120 in each arm accounting for an expected drop-out rate of 20%, is appropriate to obtain the desired statistical power.

Sample size was computed using STATA statistical software (StataCorp).

#### Data analyses

*Intention to treat* analysis will be adopted to account for possible issues related to noncompliance and missing outcomes. Data quality and internal consistency will be assessed *ex-post* using Cronbach’s alpha. In addition, automatic routines will be performed on the data to detect possible outliers, which will be excluded from further analyses. Explorative univariate and bivariate data analyses will then be performed.

For each study outcome, the intervention and control arm will be compared, as the difference between baseline and 12-month time-point, using independent sample t-tests. A mixed-design ANOVA will be used to compare each study outcome between intervention and control arm at the four different time-points, followed by *post-hoc* pairwise comparisons when a significant interaction is found. Participants’ characteristics (e.g., age, sex, fitness status, physical activity level, and metabolic profile) will be used as covariates, and appropriate interaction terms will be assessed and may be included in the model. For all tests, 2-sided *p* values with an α level of significance of 0.05 will be used. Bonferroni’s criterion will be used to adjust the overall α level to correct for multiple tests.

The assumptions of each statistical test will be assessed before analyses and, if not met, either data transformation will be performed or alternative statistical methods will be adopted (e.g., GEE, mixed-models, non-parametric analyses, etc.) will be performed. The residuals and goodness of fit of the models will also be examined.

SPSS Statistics (IBM) and R (R Core Team) software will be used to perform data analyses.

## Discussion

There is growing consensus on the central role of PA in maintaining overall health and quality of life. However, most T2D patients still adopt sedentary behavior and do not meet the minimum threshold of PA needed to get health benefits and manage their chronic clinical condition.

Given the prevalence of inactivity and sedentary behavior among T2D patients, strategies able to promote PA may play a critical role in public health care, and if its economic impact is minimized it is more likely that PA could be made available to patients over time and even become part of standardized treatment in clinical settings. ERSs appear to be a promising tool to help achieve this goal. Indeed, the guidance of the UK National Institute for Health and Care Excellence currently recommends that ERSs be implemented for T2D patients, even though their evidence-based effectiveness and cost-effectiveness still need further investigation to be verified [50]. Moreover, since PA is a complex behavior influenced by several factors [51] that can affect its adoption and maintenance in different ways, behavior change strategies able to increase motivation to adopt an active lifestyle are also recommended [52]. Fortunately, in the current information and communication technology era, behavior change initiatives have new tools at their disposal: indeed, emerging evidence has shown the potential of computers, smartphones, and wearables to boost the motivation to switch to, and maintain, an active lifestyle [53–56]. The use of WBAs in treatment plans for T2D patients has generated particular research interest [57]. In line with this new approach, the main novelty of the TRIPL-A study is the implementation of an ERS, supported by a multi-purpose WBA that can be accessed by physicians, exercise specialists, and patients to input data and retrieve relevant information. The WBA provides support for the exercise referral, but it is primarily designed around the patients and their needs, with the aim of motivating them to exercise, while at the same time monitoring their compliance to prescribed exercise and adherence to the overall training protocol. The inclusion of the WBA as an integral part of this trial fits nicely with the use of a discontinuously supervised training protocol, making the discontinuous nature of the protocol one of the particular strengths of the study. Indeed, the WBA has dual aims: to educate T2D patients on “how to” train in order to get the clinical benefits of exercise (supervised periods); to increase their “willingness” to exercise (non-supervised periods). Patients, in fact, have to enter the exercise parameters into the WBA at the end of each self-managed training session. Hence, the actual adherence of the patients to the training protocol is readily available to the exercise specialists and physicians, who can monitor the patients and encourage them to exercise effectively. Furthermore, interrupting training supervision reduces the burden associated with the ERS in terms of personnel and facility costs. This is of paramount importance for the healthcare systems that have to manage limited budgets and a growing number of older T2D patients.

Finally, the ERS and the WBA of the TRIPL-A are specifically designed to provide support for the Italian primary care settings, aiming to enhance the partnership among the stakeholders involved in the current management of the clinical conditions of T2D patients. The lack of coordination among stakeholders is a widely acknowledged weak point in the Italian framework. To our knowledge, this is the first RCT to be carried out with the aim of evaluating the effectiveness of an ERS in Italian primary care settings. Moreover, the results of this study will add data to the scant literature regarding exercise training, PA promotion and engagement, sedentary behavior and sitting time, and the management of clinical conditions in older T2D patients.

In conclusion, the TRIPL-A study described herein could pave the way for new healthcare approaches in the prevention and treatment of T2D. The study is based on maximizing the potential of ERS, which could become part of the routinely prescribed interventions (particularly when supported by ad-hoc WBAs), and on shifting the focus of disease management to patient’s personal empowerment, the starting point for the development of new approaches to health care.

## Abbreviations

%HRR: Heart rate reserve percentage
%HR_target_: Target heart rate percentage
BMI: Body mass index
CR-10: 10-point category ratio scale
CRF: Case report form
DBP: Diastolic blood pressure
EQ-5D-5L: Euro Quality of Life 5 Dimensional questionnaire
ERS: Exercise referral scheme
GP: General practitioner
HR: Heart rate
HR_max_: Maximum heart rate
HRR: Heart rate reserve
HR_rest_: Resting heart rate
HR_target_: Target heart rate
IPAQ: International Physical Activity Questionnaire
LDCW: Long distance corridor walk
MET: Metabolic equivalent
PA: Physical activity
PSQI: Pittsburgh Sleep Quality Index
SBP: Systolic blood pressure
T2D: Type 2 diabetes
*V̇*O_2max_: Maximal oxygen uptake
*V̇*O_2_R: Oxygen uptake reserve
WBA: Web-based application
WHR: Waist-to-hip ratio

## References

[1] Rice NE, Lang IA, Henley W, Melzer D (2010). Baby boomers nearing retirement: the healthiest generation?. Rejuvenation Res.

[2] World Health Organization. Global Recommendations on Physical Activity for Health. WHO, editor. Geneva: World Health Organization; 2010. 26180873

[3] American Diabetes Association (2015). Foundations of care: education, nutrition, physical activity, smoking cessation, psychosocial care, and immunization. Sec. 4. In Standards of Medical Care in Diabetesd-2015. Diabetes Care.

[4] American Diabetes Association (2015). Standards of medical care in diabetes-2015: summary of revisions. Diab Care.

[5] Henson J, Dunstan DW, Davies MJ, Yates T (2016). Sedentary behaviour as a new behavioural target in the prevention and treatment of type 2 diabetes. Diab Metab Res Rev.

[6] Dempsey PC, Larsen RN, Sethi P, Sacre JW, Straznicky NE, Cohen ND (2016). Benefits for type 2 diabetes of interrupting prolonged sitting with brief bouts of light walking or simple resistance activities. Diab Care.

[7] Baker PR, Francis DP, Soares J, Weightman AL, Foster C (2011). Community wide interventions for increasing physical activity. Cochrane Database Syst Rev.

[8] Baker PR, Francis DP, Soares J, Weightman AL, Foster C (2015). Community wide interventions for increasing physical activity. Cochrane Database Syst Rev.

[9] Kennerly A-M, Kirk A (2018). Physical activity and sedentary behaviour of adults with type 2 diabetes: a systematic review. Prac Diab.

[10] Coonrod BA, Betschart J, Harris MI (1994). Frequency and determinants of diabetes patient education among adults in the U.S. population. Diabetes Care.

[11] Lorig K, Ritter PL, Villa FJ, Armas J (2009). Community-based peer-led diabetes self-management: a randomized trial. Diab Educ.

[12] Domenech MI, Assad D, Mazzei ME, Kronsbein P, Gagliardino JJ (1995). Evaluation of the effectiveness of an ambulatory teaching/treatment programme for non-insulin dependent (type 2) diabetic patients. Acta Diabetol.

[13] Lohmann H, Siersma V, Olivarius NF (2010). Fitness consultations in routine care of patients with type 2 diabetes in general practice: an 18-month non-randomised intervention study. BMC Fam Pract.

[14] Avery L, Flynn D, van Wersch A, Sniehotta FF, Trenell MI (2012). Changing physical activity behavior in type 2 diabetes: a systematic review and meta-analysis of behavioral interventions. Diabetes Care.

[15] Rabin BA, Brownson RC, Kerner JF, Glasgow RE (2006). Methodologic challenges in disseminating evidence-based interventions to promote physical activity. Am J Prev Med.

[16] Anokye NK, Trueman P, Green C, Pavey TG, Hillsdon M, Taylor RS (2011). The cost-effectiveness of exercise referral schemes. BMC Public Health.

[17] Pavey TG, Taylor AH, Fox KR, Hillsdon M, Anokye N, Campbell JL (2011). Effect of exercise referral schemes in primary care on physical activity and improving health outcomes: systematic review and meta-analysis. BMJ.

[18] Pavey TG, Anokye N, Taylor AH, Trueman P, Moxham T, Fox KR (2011). The clinical effectiveness and cost-effectiveness of exercise referral schemes: a systematic review and economic evaluation. Health Technol Assess.

[19] Elley CR, Kerse N, Arroll B, Robinson E (2003). Effectiveness of counselling patients on physical activity in general practice: cluster randomised controlled trial. BMJ.

[20] Lewis BS, Lynch WD (1993). The effect of physician advice on exercise behavior. Prev Med.

[21] Newton RL, Han H, Stewart TM, Ryan DH, Williamson DA (2011). Efficacy of a pilot internet-based weight management program (H.E.a.L.T.H.) and longitudinal physical fitness data in Army reserve soldiers. J Diabetes Sci Technol.

[22] Turner-McGrievy G, Tate D (2011). Tweets, apps, and pods: results of the 6-month Mobile pounds off digitally (Mobile POD) randomized weight-loss intervention among adults. J Med Internet Res.

[23] Cotter AP, Durant N, Agne AA, Cherrington AL (2014). Internet interventions to support lifestyle modification for diabetes management: a systematic review of the evidence. J Diabetes Complicat.

[24] Jennings CA, Vandelanotte C, Caperchione CM, Mummery WK (2014). Effectiveness of a web-based physical activity intervention for adults with type 2 diabetes-a randomised controlled trial. Prev Med.

[25] American Diabetes Association (2015). Classification and diagnosis of diabetes. Diab Care.

[26] Bull FC, Armstrong TP, Dixon T, Ham S, Neiman A, Pratt M. Physical inactivity. In: Ezzati M, Lopez AD, Rodgers A, Murray CJL, editors. Global and regional burden of diseases attributable to selected major risk factors. Comparative quantification of health risks: global and regional burden of disease attributable to selected major risk factors. 1. [Geneva]: World Health Organization; 2004. p. 743.

[27] Health Canada - Health Services and Promotion Branch. The handbook for Canada’s physical activity guide to healthy active living. Ottawa: Canadian Society for Exercise Physilogy; 1998.

[28] Chodzko-Zajko WJ, Proctor DN, Fiatarone Singh MA, Minson CT, Nigg CR, American College of Sports Medicine (2009). American College of Sports Medicine position stand. Exercise and physical activity for older adults. Med Sci Sports Exerc.

[29] Chodzko-Zajko WJ. ACSM's exercise for older adults. 1^st^ ed. Philadelphia: Wolters Kluwer/Lippincott Williams & Wilkins; 2014. p. 236.

[30] Garber CE, Blissmer B, Deschenes MR, Franklin BA, Lamonte MJ, Lee IM (2011). American College of Sports Medicine position stand. Quantity and quality of exercise for developing and maintaining cardiorespiratory, musculoskeletal, and neuromotor fitness in apparently healthy adults: guidance for prescribing exercise. Med Sci Sports Exerc.

[31] Colberg SR, Albright AL, Blissmer BJ, Braun B, Chasan-Taber L, Fernhall B (2010). Exercise and type 2 diabetes: American College of Sports Medicine and the American Diabetes Association: joint position statement. Exercise and type 2 diabetes. Med Sci Sports Exerc.

[32] Colberg SR, Sigal RJ, Fernhall B, Regensteiner JG, Blissmer BJ, Rubin RR (2010). Exercise and type 2 diabetes: the American College of Sports Medicine and the American Diabetes Association: joint position statement. Diabetes Care.

[33] Hordern MD, Dunstan DW, Prins JB, Baker MK, Singh MA, Coombes JS (2012). Exercise prescription for patients with type 2 diabetes and pre-diabetes: a position statement from exercise and sport science Australia. J Sci Med Sport.

[34] Pescatello LS (2014). American College of Sports Medicine. ACSM's guidelines for exercise testing and prescription. 9^th^ ed.

[35] Gellish RL, Goslin BR, Olson RE, McDonald A, Russi GD, Moudgil VK (2007). Longitudinal modeling of the relationship between age and maximal heart rate. Med Sci Sports Exerc.

[36] Karvonen MJ, Kentala E, Mustala O (1957). The effects of training on heart rate; a longitudinal study. Ann Med Exp Biol Fenn.

[37] Swain DP (2014). American College of Sports Medicine. ACSM's resource manual for guidelines for exercise testing and prescription. 7^th^ ed.

[38] Borg G. Borg's Perceived exertion and pain scales. Champaign, IL: Human Kinetics; 1998. viii, 104 p. p.

[39] Klaren RE, Hubbard EA, Motl RW (2014). Efficacy of a behavioral intervention for reducing sedentary behavior in persons with multiple sclerosis: a pilot examination. Am J Prev Med.

[40] Masala D, Mannocci A, Unim B, Del Cimmuto A, Turchetta F, Gatto G (2012). Quality of life and physical activity in liver transplantation patients: results of a case-control study in Italy. Transplant Proc.

[41] Segura-Jimenez V, Munguia-Izquierdo D, Camiletti-Moiron D, Alvarez-Gallardo IC, Ortega FB, Ruiz JR (2013). Comparison of the International Physical Activity Questionnaire (IPAQ) with a multi-sensor armband accelerometer in women with fibromyalgia: the al-Andalus project. Clin Exp Rheumatol.

[42] Tomioka K, Iwamoto J, Saeki K, Okamoto N (2011). Reliability and validity of the international physical activity questionnaire (IPAQ) in elderly adults: the Fujiwara-kyo study. J Epidemiol.

[43] Tran DV, Lee AH, Au TB, Nguyen CT, Hoang DV (2013). Reliability and validity of the international physical activity questionnaire-short form for older adults in Vietnam. Health Promot J Austr.

[44] Simonsick EM, Fan E, Fleg JL (2006). Estimating cardiorespiratory fitness in well-functioning older adults: treadmill validation of the long distance corridor walk. J Am Geriatr Soc.

[45] Simonsick EM, Montgomery PS, Newman AB, Bauer DC, Harris T (2001). Measuring fitness in healthy older adults: the health ABC long distance corridor walk. J Am Geriatr Soc.

[46] Thiel DM, Al Sayah F, Vallance JK, Johnson ST, Johnson JA (2017). Association between physical activity and health-related quality of life in adults with type 2 diabetes. Can J Diabetes.

[47] Curcio G, Tempesta D, Scarlata S, Marzano C, Moroni F, Rossini PM (2013). Validity of the Italian version of the Pittsburgh sleep quality index (PSQI). Neurol Sci.

[48] Telford O, Diamantidis CJ, Bosworth HB, Patel UD, Davenport CA, Oakes MM, et al. The relationship between Pittsburgh sleep quality index subscales and diabetes control. Chronic Illn. 2018;1742395318759587. https://www.scopus.com/record/display.uri?eid=2-s2.0-85042439107&origin=inward&txGid=1ae7a66758659ea7685065fdf9010c74. 10.1177/1742395318759587 PMC7187808 29466873

[49] Heyward VH, Gibson AL (2014). Advanced fitness assessment and exercise prescription.

[50] National Institute of Health and Clinical Excellence (NICE). Exercise referral schemes to promote physical activity; NICE public health guidance 54. London: National Institute for Health and Care Excellence; 2014.

[51] Bauman AE, Sallis JF, Dzewaltowski DA, Owen N (2002). Toward a better understanding of the influences on physical activity: the role of determinants, correlates, causal variables, mediators, moderators, and confounders. Am J Prev Med.

[52] National Institute for Health and Care Excellence (NICE). Behaviour change: individual approaches; NICE public health guidance 49 London: National Institute for Health and Care Excellence; 2014.

[53] Bort-Roig J, Gilson ND, Puig-Ribera A, Contreras RS, Trost SG (2014). Measuring and influencing physical activity with smartphone technology: a systematic review. Sports Med.

[54] Rowsell A, Muller I, Murray E, Little P, Byrne CD, Ganahl K (2015). Views of people with high and low levels of health literacy about a digital intervention to promote physical activity for diabetes: a qualitative study in five countries. J Med Internet Res.

[55] Morton K, Dennison L, May C, Murray E, Little P, McManus RJ (2017). Using digital interventions for self-management of chronic physical health conditions: a meta-ethnography review of published studies. Patient Educ Couns.

[56] Morrison LG, Hargood C, Lin SX, Dennison L, Joseph J, Hughes S (2014). Understanding usage of a hybrid website and smartphone app for weight management: a mixed-methods study. J Med Internet Res.

[57] ISRCTN Registry. A multi-centred randomised trial to assess if adding web-based support to exercise referral schemes for individuals with metabolic, musculo-skeletal and mental health conditions can increase physical activity after 12 months: ISRCTN Registry; 2017 [Available from: http://www.isrctn.com/ISRCTN15644451].

